# Future of Regional Anaesthesia: “A Block for Everyone”

**DOI:** 10.5152/TJAR.2023.22101

**Published:** 2023-04-01

**Authors:** Yavuz Gürkan, Kamen Vlassakov

**Affiliations:** 1 Department of Anaesthesiology, Koç University Hospital, Istanbul, Turkey; 2 Department of Anaesthesiology, Perioperative and Pain Medicine, Brigham and Women’s Hospital, Harvard Medical School, Boston, Massachusetts, USA

Regional anaesthesia practice has changed dramatically, especially in the last 2-3 decades. Anaesthesiologists are far beyond doing only extremity and/or central neuraxial blocks. Unquestionably, the introduction of ultrasound into regional anaesthesia practice has a pivotal role in the so-called “renaissance” of regional anaesthesia today. Ultrasound allowed us to see not only the nerves themselves but also the fascial planes where nerves are located. In our own clinical practices, the diversity of the blocks is nothing comparable to what we used to do 15-20 years ago. Besides doing randomised controlled studies to test the newly introduced blocks, we often publish about selective blocks as case reports or short editorials just to inform the anaesthesia community, to let them know that all these different blocks are technically feasible and patients benefit a lot from receiving these selective blocks. Moreover, we need to recognise that with ultrasound guidance and improved understanding of sonoanatomy, new techniques to deliver local anaesthetics to target nerves will inevitably emerge ad hoc in expert hands with or without scientific publications.

Today, we are discussing with Dr K.V. from Harvard University some essential issues about the future of regional anaesthesia, trying to answer the question whether there will be a block for every single patient entering the operating theatres to provide anaesthesia or analgesia or some benefits beyond anaesthesia.

## Do you think ESPB has changed the practice of regional anaesthesia and will it survive the test of time?

Y.G. Erector Spina Plane Blocks (ESPB) is still a very young block introduced in 2016. There are thousands of publications trying ESPB almost for any indication in the operating room. Today, it is an integral part of our anaesthesia practice for breast surgery and included in the armamentarium of anaesthesiologists for thoracic surgery. It has always been challenging to perform regional anaesthesia for cardiac surgery due to anticoagulant use in the peri-operative period. Blocks like ESPB now offer an option for providing perioperative analgesia for cardiac surgery patients. For paediatric age group, ESPB has been shown to be very effective for both thoracic and abdominal surgeries. ESPB offered an option for spine surgery to reduce opioid requirement. I personally think ESPB is more effective in thoracic than lumbar levels and in the same manner in paediatrics compared to adults. This information comes from our extensive clinical experience with ESPB in different clinical scenarios. Generally speaking, ESPB has been a very successful adjunct to anaesthesia practice for many anaesthesiologists all over world due to technical simplicity and safety.

K.V. OK—let’s make this discussion more provocative… Yes, this may be a new block technique by designation and scientific interest, but to challenge the audience, it is not really a new block by injectate location/distribution. In fact, the majority of paravertebral blocks (before or after ultrasound guidance) that fail to reach their intended target—the proper paravertebral space, deliver inadvertently the local anaesthetic mixture into planes, currently described and studied as retrolaminar plane, erector spinae plane, mid-point, even TLIP in the lumbar region. And it has been shown that injections of local anaesthetics in those interfascial planes also provide analgesia—how lucky for us and our patients! The new techniques mentioned above strive to explain, explore, and exploit the mechanisms of these fortunately beneficial effects… And, should we designate these blocks as truncal interfascial plane blocks or “paravertebral by proxy” or just “failed paravertebral blocks”?! Or should we accept ESPB to be a representative of the generic “any fascial plane blocks” (AFPBs) that I introduce my mentees to with loving respect and a mockingly optimistic chuckle, illustrating the ever-increasing number of reported interfascial blocks.

Practically now: (1) ESPB is one of the new plane blocks, very similar to the retrolaminar block anatomically, that are shown to produce trunk analgesia with minimal risks, even in anti-coagulated patients; (2) this block is relatively easy to learn, teach and perform even in patients with challenging anatomy; (3) it necessitates large amounts of local anaesthetics and their systemic effects should not be ignored as a significant contributing mechanism, especially in the absence of exhaustive research, investigating that aspect of large-dose fascial plane blocks; (4) ESPB delivers local anaesthetics farther away from its intended anatomical target—the spinal nerves, than a paravertebral or a proximal intercostal block; therefore, its analgesic effects would be predictably inferior to the latter techniques (one exception would be ESPB or retrolaminar block for spine surgery, where the dorsal rami are the primary target); (5) ESPB is a very useful technique in our armamentarium, especially when better targeted techniques are contraindicated or anatomically difficult—for example, we often use ESPB as a first block technique in patients with multiple rib fractures or post-thoracotomy pain who are coagulopathic, then transition to “deeper” block techniques as needed and when safe.

## Do you think interfascial plane blocks will replace central neuraxial blocks?

Y.G. It is for sure that interfascial plane blocks will constitute an alternative to central neuroaxial blocks. Spinal anaesthesia is stronger than epidural and epidural is stronger than ESPB or similar interfascial plane blocks. Today, interfascial plane blocks are an integral part of multimodal analgesia. Multimodal analgesia utilises analgesic medications and regional anaesthesia techniques together to minimise risks and complications of each technique yet improving quality and safety in the perioperative period. As part of multimodal analgesia interfascial plane blocks can be considered a reliable alternative to epidurals. Epidural anaesthesia will survive. Yet more and more anaesthesiologists will be using these new interfascial plane blocks. Time will show us the advantages and limitations of each technique.

K.V. As a devoted regionalist, I would not want one good block technique to replace or “eliminate” another one, especially when it comes to methods that have more than a century old record of proven patient benefits and safety. The greater the diversity of instruments in our toolbox, the better our versatility and ability to help every patient, and the higher the chance we can successfully optimise and even personalise patient care. In a case where neuraxial anaesthesia/analgesia is contraindicated or very difficult, interfascial plane blocks provide a very good alternative. They can also be used as a temporising measure till either neuraxial techniques are feasible or “aggressive” analgesia is no longer needed. Similar “staging” of different regional techniques with different purposes should be the core of multimodal analgesia.

For example, a spinal anaesthetic could provide the primary surgical anaesthesia for Caesarean section, while a long-acting or continuous interfascial plane blocks should provide the post-operative analgesia in conjunction with other multimodal agents and techniques. However, one cannot underestimate or undermine the proven efficacy and potency of long-acting neuraxial opioids, such as intrathecal morphine. So instead of looking for a competition, we should rather aim at synergism.

## To what extent does RA contribute to prevent the opioid crisis?

Y.G. Today, medicine values regional anaesthesia to a great extend for one or other reason. Either contributing to solve the problem of peri-operative opioid crisis or facilitating ERAS protocols almost every anaesthesia protocol ends up with the conclusion that regional anaesthesia practices should be an integral part of anaesthesia care for all patients undergoing surgery. Thinking of chronic pain patients or opioid users for any reason, these patients are not tolerant to local anaesthetics. Regional anaesthesia is probably the most important tool to solve the acute peri-operative pain problem of chronic pain patients.

K.V. Regional anaesthesia/analgesia is clearly the most important and the most logical tool we have in treating and preventing acute (and chronic) pain, and the related cases of opioid overuse and addiction. Clearly, not all opioid addiction stems from peri-operative pain or other iatrogenic factors, by far. And we, regionalists, might yet play some unexpected roles in treating such addiction—why not sympathetic blocks in withdrawal?

In our everyday lives, we should start by optimising our nerve block-focused and patient-tailored multimodal analgesia, then learn more about the pain trajectories, match and dynamically adapt our strategies, minimse opioid use but also educate patients and health-care workers (starting with our surgical colleagues) about the unquestionable benefits of regional anaesthesia. Cultivating realistic expectations in our patients and colleagues should also come with decreased production pressures and misplaced perceptions of nerve blocks as causes of unnecessary delays in a collaborative culture that puts the patient first.

## How shall we improve the safety of RA practice?

Y.G. Ultrasound has already provided a visual monitor or a safety measure during nerve blocks. Image quality that means resolution of ultrasound devices has improved dramatically. I am sure that image quality will keep improving in time and will provide further detailed and clear image of our neural targets for regional anaesthesia. Using ultrasound guidance, the incidence of LAST has decreased significantly. The incidence of neurologic complications is also decreasing. Scientifically, it is difficult to conduct studies when true incidence of RA associated complications is already very low. Pressure monitoring and dual control *via* the use of nerve stimulators to verify the neural target and to help us avoid intra-neural injections also contributes to reducing the incidence of neurological complications. Technical skills and vigilance of anaesthesiologist is probably the most important component of patient safety during regional anaesthesia. Understanding the philosophy of regional anaesthesia is important when we have a large scale of different regional anaesthesia techniques. It is very important to know the limitations and contraindications of each technique.

K.V. It is very hard to demonstrate further improvement in a very safe environment and with procedures that have been consistently associated with remarkable safety records. The future of regional anaesthesia is bright and continuing advances in safety are all but guaranteed with innovations that we now take for granted—direct real-time needle guidance with ever improving image quality, pre-procedural safety time-outs, mandatory peri-procedural monitoring, modern comprehensive provider education and training, including simulation. So the goals and effective trends have been already set to be followed well with vigilance and flexibility accounting for future developments.

At the same time, the virtual explosion of regional anaesthesia applications and use, overflowing into non-standard locations and circumstances and often even beyond the stereotypical care providers, will pose new challenges. Our role as specialty-trained regional anaesthesiologists should expand into consultants who help set up standards of care, including monitoring, provider training and procedural safety, and even expert back up. Such roles will inevitably provoke wide debates and should be defined in constructive collaboration. The inclusion of high-tech solutions and/or aids through machine-learning and artificial intelligence is still nascent, but we should not let ourselves be (over)taken by surprise.

## What are your block choices for cardiac and thoracic surgery? Do you think there are winds of change?

Y.G. Epidural anaesthesia has been utilised in cardiac surgery patients to provide both surgical anaesthesia and analgesia. Epidural analgesia has been used for patients with refractory angina pectoris patients who are poor candidates for surgery. The main limitation of thoracic epidurals for cardiac surgery patients is the use of heparin and the potential risk of epidural hematoma. Spinal cord injury also had devastating complications following thoracic epidural attempts. Therefore, in clinical practice anaesthesiologists refrain from using epidural anaesthesia/analgesia at thoracic level.

Today, interfascial plane blocks provide a true alternative to thoracic epidurals. Yet interfascial plane blocks will fail to provide surgical anaesthesia for cardiac surgery patients. Yet relatively minor thoracic surgeries with iv sedation has been reported.

Transversus Thoracic Muscle Plane Block presents an anterior approach to block thoracic segments in supine position. It has been used for both paediatric and adult cardiac surgery patients. There is already accumulated experience with pectoral blocks and serratus plane blocks for thoracic surgery patients. Although thoracic paravertebral block is almost 100 years old block, it is deeper and technically more advanced block compared to more superficial blocks like ESPB. Our clinical experience proves that ESPB provides coronary dilatation, decreases opioid requirement and reduces the incidence of post-operative arrhythmias.

K.V. Indeed, under rare circumstances, providing surgical anaesthesia with neuraxial techniques is possible. In my opinion however, such methods cannot become the standard of care, neither would they ever be widely applicable or acceptable for patients and providers. Not till cardiac surgery becomes truly minimally invasive, similar to the percutaneous transcatheter valve replacement techniques. And in those instances, regional anaesthesia can be employed to the access site and sources of nociceptive stimulation—peripheral nerve blocks and interfascial plane blocks being most appropriate.

Yet, cardiac surgery is associated with significant and prolonged post-operative pain that can and should be treated better, and the various trunk block techniques seem best suited from effectiveness and safety standpoint. This is a new field and the jury would likely be out for a few more years, regarding to what the best-suited blocks in each patient/procedure are. In my practice and anecdotal experience, the best technique for cardiac surgery *via* mini-thoracotomy is the continuous *proximal intercostal block* and the technique best suited (in our hands) for median sternotomy is the continuous bilateral *parasternal subpectoral plane block* (aka pecto-intercostal plane block).

Our proximal intercostal blocks are performed 1-2 cm lateral to the lateral tip of the transverse process of the corresponding vertebra under ultrasound guidance. Delivering the local anaesthetic between the internal intercostal membrane and the endothoracic fascia/parietal pleura complex at that level, then threading a continuous block catheter no more than 5 cm beyond the needle tip virtually guarantees that the needle and catheter would not access (or cause bleeding inside) the spinal canal and the neuraxis in these patients who are anti-coagulated before, during or/and after surgery. Its clinical effects are hard to distinguish from the more commonly used paravertebral injection, while the entry is further removed from the neuraxis and the visualisation of relevant sonoanatomy (especially the pleura) is typically significantly better. We place 2 catheters—one corresponding to the level of the thoracotomy incision and another 2-3 levels caudally to augment the thoracic wall analgesia and to cover the chest tube site.

The continuous bilateral parasternal subpectoral plane block is performed by us at the end of surgery after complete wound closure where the plane between the pectoral muscle and its fascia on one side and the rib cartilages and external intercostal membrane is gradually hydro-dissected with dilute local anaesthetic under US guidance and followed with the block needle over 4-5 ribs. Then, a multiport catheter is laid in the created space to be infused with diluted local anaesthetic for the first 4-5 post-operative days. This procedure is performed on both sides and in very close proximity of the sternum.

As most thoracic surgery patients are not anti-coagulated, our main analgesia method for thoracotomy remains epidural analgesia, while the continuous proximal intercostal blocks (with paravertebral, retrolaminar and erector spinae plane blocks in reserve) are utilised in patients with challenging or failed epidural blocks or in those cases where neuraxial techniques are contraindicated.

## Do you think that it is feasible to do breast surgery under nerve blocks alone?

Y.G. Breast surgery under regional anaesthesia has always been a challenge for the anaesthesiologist and paravertebral blocks are the most famous blocks for this kind of surgery. Today, we have many more options including pectoral blocks, rhomboid intercostal block and ESPB. My experience and detailed analysis of the literature show that breast innervation is rather complex and is supplied by at least 3 nerve groups namely cervical plexus, pectoral plexus and thoracic spinal nerves. Understanding the anatomy is the key for success and also helps us understand why regional anaesthesia techniques fail to provide complete anaesthesia for breast surgery. All previous experiences including our studies have shown that patients need some degree of additional sedation besides these interfascial plane blocks. My favourite approach is still paravertebral block plus pectoral blocks for surgical anaesthesia of breast tissue. For providing analgesia alone our favourite approaches include ESPB and rhomboid blocks for the sake of simplicity and safety.

K.V. The complexity of the innervation of the breast is remarkable and providing complete surgical anaesthesia for mastectomy can be quite challenging, yet doable, given sufficient expertise and time for the blocks to work, surgical areas to be tested and certain supplemental blocks to be performed. The paravertebral blocks and epidural anaesthesia have been the most predictable surgical anaesthesia techniques—the role of cervical plexus branches remains variable, but quite manageable with supplemental blocks. In our hands, the proximal intercostal blocks have proven equivalent to paravertebral blocks in providing superb analgesia and also surgical anaesthesia for breast surgery—the latter being also dependent on surgeon’s and patient’s preference and acceptance of proceeding without general anaesthesia. The psychological aspects of mastectomy surgery (“breast amputation”) cannot be understated and appropriate sedation (including amnesia) and emotional support remain critical. For analgesia alone, the whole armamentarium of paravertebral and proximal intercostal blocks, and interfascial plane blocks from pectoral blocks, serratus anterior blocks, erector spinae and retrolaminar blocks have been employed by our team. It must be said that well-targeted local infiltration by surgeons who in our institution have carefully observed and learned from our interfascial plane techniques have similar benefits and becoming increasingly difficult to eclipse, especially for smaller breast surgery interventions, such as lumpectomy or partial resections.

## How patients undergoing laparoscopic/robotic surgery can benefit from RA techniques?

Y.G. Contrary to the belief that laparoscopic surgery results in less pain compared to open surgery, patients experience severe pain at trocar entry sites and surgical material removal sites. Visceral pain and effects of pneumo-peritoneum also contribute to post-operative discomfort. Regional anaesthesia techniques can help to relieve this pain and discomfort of the patients. Anaesthesiologists have to choose the correct technique to obtain the optimum balance of function and analgesia. Epidural anaesthesia/analgesia may be overtreatment for these patients. Literature and our own clinical experience proves that newly introduced interfascial plane blocks may provide analgesia without compromising motor functions and avoiding potential risks of central neuro-axial blocks. We have earlier conducted studies on the use of paravertebral blocks for cholecystectomy, and today, we are using many different approaches such as TAP, rectus sheath, transversalis fascia plane and external oblique intercostal blocks. An important reminder is that these blocks should be part of the multimodal analgesia approach. We should include systemic analgesics for the optimum analgesia and patient comfort.

K.V. The combination of minimal abdominal wall pain, relatively easy to address by adequate surgical infiltration and often substantial visceral pain, often described as distention, spasm or colic, is challenging, and should be addressed with multimodal approach that may include not only regional anaesthesia but also spasmolytic and anti-inflammatory agents if not contraindicated. Regional anaesthesia techniques, including truncal interfascial plane blocks, such as quadratus lumborum and ESP blocks, as well as the more invasive paravertebral and proximal intercostal blocks that may provide superior abdominal wall and visceral analgesia should be considered in the context of relative risks and benefits. We must also recognise that the reported and observed in our practice benefits of high-dose interfascial plane blocks in such circumstances may be (at least in part) a manifestation of the beneficial systemic effects of local anaesthetics absorbed into the bloodstream.

Additionally, we currently have very limited (if any) means of identifying and predicting which patient would do well with trivial multimodal medications and which would need everything we have in our regional anaesthesia armamentarium. We need to advance our work in creating tools that would direct our personalised care based on psychosocial (e.g. catastrophising) and neurophysiological (e.g. pain thresholds and mini-quantitative sensory testing) characteristics.

## How RA is changing for paediatric surgery patients? Do we still need a caudal block?

Y.G. Caudal anaesthesia was the main and only regional anaesthesia technique performed by many anaesthesiologists for paediatric patients. Today, there is almost no age limit for performing nerve blocks in children. The hesitation of performing nerve blocks under general anaesthesia is over as long as regional anaesthesia techniques are used properly. Orthopaedic patients already benefit from a variety of upper and lower extremity blocks. We perform lumbar plexus block *via* Shamrock approach for paediatric hip surgery patients. Penile nerve block is a simple yet clinically effective technique for treating pain of penile surgery. Erector spinae plane block had great success for providing analgesia of abdominal and thoracic surgeries. Many other interfascial plane blocks can be utilised in paediatric patients too.

K.V. I would refrain from strong opinions about regional anaesthesia in the paediatric anaesthesia world. Yet, from the literature and vicariously through my colleagues and family who work in that specialty area, it seems that the impact of ultrasound-guided nerve blocks has been even greater in the care of paediatric patients. Significant improvements in safety and regional anaesthesia acceptance, as long as the ubiquitous shift from neuraxial to peripheral to interfascial plane blocks is evident in this field as well, providing a multitude of options and improved perioperative care for the youngest patients. One could also hypothesise that the interfascial plane blocks (similar to the older fascia iliac block technique, originally described in children) might be more anatomically predictable in children when it comes to injectate spread within or beyond a fascial layer. Indeed, regional anaesthesia in the paediatric patient nowadays looks significantly more similar to adult regional anaesthesia than it did a couple of decades ago, mostly due to ultrasound guidance.

## Do you think that the blocks we are performing prevent chronic pain development after surgery?

Y.G. Development of post-surgical chronic pain is major health problem affecting around 10%-20% of patients undergoing surgery. Risk factors include type of surgery, anaesthesia, preoperative pain levels and opioid use and personal psychological status of the patient. Certain patient groups such as amputation, mastectomy, thoracotomy, cardiac surgery and inguinal hernia patients have a high incidence of chronic pain reaching up to 50%-60% after surgery. The duration of the severe acute post-operative pain is a constant future of chronic pain. Inadequately treated post-surgical pain leads to central sensitisation and results in exaggerated response to painful stimuli. Regional anaesthesia definitely contributes to reducing the incidence of chronic pain yet because the pathophysiology of chronic pain is multifactorial it will not totally eliminate chronic pain problem. Providing adequate analgesia *via* use of nerve blocks will contribute to lower the number of chronic opioid users after surgery and help solve the opioid crisis all over the world.

K.V. The recognition of persistent post-surgical pain is relatively new and we still have much to learn about its characteristics and prevention. We ought to renew our interest in the con­cept of pre-emptive and preventive analgesia, as well as the study of pain trajectories after intended or unintended injury. As mentioned before, we should also develop means to pre­dict whether a patient is at higher risk for developing severe and persistent post-surgical pain, based on their psychosocial and neurophysiological characteristics in conjunction with our understanding of the variable, but typically predictable extend of surgical injury.

Our limited and hard-fought experiences with post-amputation pain prevention and treatment bring new understanding and hope for better prophylaxis and therapy in which peripheral nerve block techniques will be central and might also include ultrasound-guided peripheral neuro-stimulation. The promise is great that such techniques will also lead to a significant decrease in injury/surgery-related opioid overuse and addiction.

## Do you think that every patient entering the OR will have a certain block for surgery and/or analgesia?

Y.G. There are some important milestones in the field of regional anaesthesia. Nerve stimulation used in 1960s and introduction of ultrasound have allowed the great majority of anaesthesiologists to perform nerve blocks in their daily life with improving success rates and safety. Now, we have another new step which is the use of interfascial plane blocks and performing very selective nerve blocks for surgery and/or analgesia. When readers review our publications they can see that we are performing a wide variety of regional anaesthesia performances ranging from dorsal penile nerve block to selective greater auricular nerve block besides all the known the interfascial plane blocks. Today, we are performing a block almost for any patient entering the operating rooms and the one who do not get a selective nerve block remains to be the exception.

K.V. Yes, indeed—until surgery can become so minimally invasive that it can neither trigger a significant stress response nor an actionable nociceptive stimulation, our maximalist goal should be to abolish those with a well-targeted and safe regional anaesthesia technique. The evolution of our filed has already provided lots of viable options and will continue to do so, based on evolving understanding of relevant anatomy and physiology, as well as clinical logic and meaningful outcomes. I often joke with apparent pride that “if there is a nerve, there is a block” and that with newer ultrasound-guided techniques, “even if there isn’t a nerve… there is a block.” Our patients’ well-being and enhanced recovery provide the ultimate verdict and praise of our dedicated efforts.

## What do you think about “regional anaesthesia outside the box” applications?

Y.G. Regional anaesthesia has benefits beyond simply treating acute pain. Regional anaesthesia will continue to lower the incidence of post-surgical chronic pain. Treating acute pain very effectively will lead to early discharge of patients from the hospital unless there are surgical contraindications. Early discharge is an integral part of any ERAS protocol. By suppressing exaggerated immune response to surgery, RA will contribute enhanced healing and prevent the devastating effects of exacerbated immune response to surgery. I think that there will a new era in the field of chronic pain. Certain number of chronic pain patients can definitely benefit from some of the newly discovered interfascial plane blocks. I am sure we will have new blocks introduced into regional anaesthesia practice. In the same manner, RA will be utilised much more frequently outside operating rooms like the emergency service or intensive care units.

K.V. Modern ultrasound-guided regional anaesthesia sets the stage for more than just effective pain control—it makes us unique in vivo sonoanatomy experts, whose opinion, assistance and intervention are and should be sought after in a variety of challenging cases. As diagnosticians, assessing abnormal anatomy with minimally invasive point-of-care ultrasound, we can assist and even guide intraoperative decisions. Currently, our regional anaesthesia experts are available and instrumental in consulting surgical colleagues—intra-operative ultrasound imaging in neuroma and nerve entrapment surgery, or with scarred neurovascular bundle in re-operation for thoracic outlet syndrome, are becoming increasingly common and sought after. Furthermore, treating or resetting (patho)physiology with sympathetic blocks such as stellate ganglion and high proximal intercostal blocks in acute clinical situations such as ventricular tachycardia electrical storms, as well as intractable angina pain, providing procedural anaesthesia/analgesia for multiple out-of-the-OR indications and creative nerve block solutions in the critically ill—these are just some of the areas our regional anaesthesiology team has been active in. It is all open for us to develop and adapt, provide and incorporate into clinical practice and standards of care.

